# Use of colloids and crystalloids for perioperative clinical infusion management in cardiac surgery patients and postoperative outcomes: a meta-analysis

**DOI:** 10.1186/s13741-024-00445-0

**Published:** 2024-07-24

**Authors:** Shan-Dong Chen, Yu-Tong Ma, Hui-Xia Wei, Xin-Rong Ou, Jia-Yi Liu, Ya-Lan Tian, Chao Zhang, Yun-Jin Xu, Yao Kong

**Affiliations:** 1 Center for Evidence-Based Medicine and Clinical Research, Taihe Hospital, Hubei University of Medicine, No.32, Renmin South Road, Shiyan, 442000 Hubei China; 2 Department of Anesthesia, Taihe Hospital, Hubei University of Medicine, No.32, Renmin South Road, Shiyan, 442000 Hubei China; 3 Department of Pediatric, Taihe Hospital, Hubei University of Medicine, No.32, Renmin South Road, Shiyan, 442000 Hubei China; 4 Department of Spine, Taihe Hospital, Hubei University of Medicine, No.32, Renmin South Road, Shiyan, 442000 Hubei China

## Abstract

**Background:**

The optimal fluid management strategy for patients undergoing cardiac surgery was controversial regarding fluid volume and intraoperative fluid types. This study aimed to assess the correlation between colloids and crystalloids used for perioperative fluid therapy in cardiac surgery patients and postoperative prognosis.

**Methods:**

The Ovid MEDLINE(R) ALL, Embase, and Cochrane Central Register of Controlled Trials databases were searched for eligible studies on fluid management strategies using colloids and crystalloids for cardiac surgery patients published before August 25th, 2023.

**Results:**

Ten randomized controlled trials met the eligibility criteria. Compared to the use of crystalloids, the use of colloids, including hydroxyethyl starch (HES), albumin, and gelatine, did not show any differences in mortality, transfusion, acute kidney injury, and atrial fibrillation rates, postoperative blood loss, the length of hospital stay, or the length of intensive care unit (ICU) stay. The results of this meta-analysis showed that the crystalloid group had significantly reduced postoperative chest tube output compared to the colloid group. In the subgroup analysis, the amount of fresh frozen plasma (FFP) infused was significantly lower when using fluid management in the ICU and when using isotonic crystalloids compared to the colloids. In addition, when using fluid management in the ICU, patients in the colloid group had a significant increase in urine volume 24 h after surgery. However, other related factors, including the type of crystalloid solution, type of colloidal solution, and timing of liquid management, did not affect most outcomes.

**Conclusion:**

Both colloids and crystalloids could be used as alternatives for perioperative fluid management after cardiac surgery. The use of crystalloids significantly reduced the postoperative chest tube output, and the need for FFP infusion decreased significantly with the use of isotonic crystalloids or fluid management during the ICU stay. ICU patients in the colloid group had higher urine output 24 h after surgery. In addition, although the infusion method was not related to most outcomes, the rates of red blood cell and FFP transfusion and postoperative blood loss in the crystalloid group seemed to be lower, which needed to be further studied in high-quality and large-sample RCTs.

**Trial registration:**

PROSPERO, CRD42023415234.

**Supplementary Information:**

The online version contains supplementary material available at 10.1186/s13741-024-00445-0.

## Introduction

With the coming of an ageing society, heart disease had become one of the most important health problems in the world. It was estimated that the number of heart disease patients will continue to increase in the coming decades (Sasayama 2008). Congenital heart disease was the most common congenital disease among newborns (Bom et al. 2011) and affected approximately five million adults in the United States (Sun et al. 2015). Although cardiac surgery reduced mortality in patients, serious and complex complications might occur during the perioperative period due to the enormous impact of surgical stimulation on patients (Vandenberghe et al. 2017) (e.g., thrombosis of the coronary artery graft, postoperative bleeding, renal failure, acute kidney injury (AKI), cerebrovascular accidents, atrial fibrillation, and seizures), and even death, the worst consequence, could occur. A study of perioperative fluid management strategies for non-cardiac surgery patients noted that inappropriate perioperative fluid management was associated with increased postoperative complications (Miller et al. 2021). It was essential to determine the optimal strategy for fluid therapy and the influence of fluid therapy on tissue oedema formation and end-organ function. However, the use of crystalloids or colloids for volume replacement in cardiac surgery patients was controversial (Bignami et al. 2017).

In 2013, the British Medical Journal (Wise 2013) published an article detailing the entire process of Boldt's falsification of data on the efficacy of hydroxyethyl starch (HES). Subsequently, nearly one hundred articles by Boldt were withdrawn, and relevant clinical evidence recommending the use of HES injection was overturned, leading to the suspension of the marketing authorization of HES injection by the European Medicines Agency (EMA). The clinical evidence that did not include that of Boldt's study should be reconstructed to provide more realistic clinical evidence for clinicians.

The common options for perioperative fluid management in cardiac surgery patients were crystalloids and colloids. Crystalloids were aqueous solutions of salts, minerals, or other water-soluble substances, such as 0.9% NaCl. Due to their low price, low rates of allergic reactions, and minor adverse effects on acid–base balance during normal dose intravenous injection (Semler and Kellum 2019), crystalloids were the fluid of choice for perioperative resuscitation and optimization in patients not requiring blood products (Garrioch and Gillies 2015). Common colloidal solutions included HES, gelatine, and albumin. Colloidal solutions had large molecular weights, and the solutions remained longer in blood vessels, providing better hemodynamics for patients, whether undergoing cardiovascular surgery (Verheij et al. 2006) or non-cardiac surgery (Santos et al. 2022). In patients with primary ovarian cancer, the infusion of blood products could be reduced to some extent by using these solutions (Feldheiser et al. 2013). But some colloidal solutions such as albumin could reduce blood coagulation ability (Rasmussen et al. 2016) and allergic reactions (J Ring KM 1977). Albumin, known for its ability to elevate plasma osmotic pressure (Fanali et al. 2012), protective effect on endothelial glycocalyx (Aldecoa et al. 2020), and reduce accumulation of interstitial fluid. However, due to its severe allergic reactions and high cost impact, its use was limited. HES was equivalent to albumin in reducing interstitial fluid accumulation during cardiac surgery (Sümpelmann et al. 2022), and compared to crystalloid solutions, the extravascular lung water index was reduced (Eising et al. 2001). For safety reasons, HES had withdrawn from the European market due to increased risks such as mortality (Martin and Bassett 2019) and AKI (Zhou et al. 2018; Lagny et al. 2016). A relevant study showed that the risk of adverse events associated with colloidal solutions supplementation was significantly increased compared with that associated with crystalloids (Ryhammer et al. 2017). In a prospective study, cardiac surgery patients did not respond better with the use of colloids than crystalloids and had an increased risk of renal replacement therapy (Bayer et al. 2013).

Due to the lack of relevant systematic evaluations, it was necessary to compare the safety and efficacy of crystalloids with those of colloids in cardiac surgery patients. This meta-analysis had systematically compared the efficacy of crystalloids and colloids in the perioperative period of cardiac surgery from the perspective of postoperative complications. We conducted a systematic meta-analysis by searching for randomized controlled trials (RCTs) that compared the use of crystalloids and colloids in cardiac surgery patients. Moreover, mortality was identified as the primary clinical outcome in this meta-analysis, with multiple secondary clinical outcomes, including the rates of transfusion, atrial fibrillation, and AKI, the length of intensive care unit (ICU) stay, urine volume within 24 h after surgery, postoperative blood loss, the length of hospital stay, and postoperative chest tube output over the first 24 h after surgery.

## Methods

The meta-analysis was developed using the Preferred Reporting Items for Systematic Reviews and Meta-analyses (PRISMA) guidelines. This systematic review and meta-analysis was registered at PROSPERO (CRD42023415234).

### Search strategy

Three electronic databases, including the Ovid MEDLINE(R) ALL, Embase, and Cochrane Central Register of Controlled Trials databases, were searched for RCTs on the influence of colloids or crystalloids on postoperative recovery after cardiac surgery procedures published up to August 25th, 2023. The following medical terms and keywords were used: cardiac surgery, heart surgery, thoracic surgery, cardiac surgical procedures, enhanced recovery after surgery, postoperative recovery, crystalloids, colloids, fluid management, fluid selection, fluid therapy, and perioperative. The detailed electronic search strategies were presented in Supplementary Method 1.

### Inclusion and exclusion criteria

Included studies were required to meet the following criteria: (1) Participants: Adults aged > 18 years undergoing cardiac surgery; (2) Intervention: Colloids, including HES, albumin and gelatine; (3) Control: Crystalloids, including iso-osmia and hypertonic crystalloids; (4) Outcomes: Rates of mortality, transfusion (including red blood cell (RBC), fresh frozen plasma (FFP), and platelet transfusions), and AKI, urine volume within 24 h after surgery, postoperative blood loss, postoperative chest tube output over the first 24 h after surgery, the length of ICU stay, and the length of hospital stay; and (5) RCTs published in English.

The exclusion criteria were as follows: (1) studies published by Boldt, (2) duplicate studies, (3) studies without data available for extraction, and (4) non-English-language studies.

### Data extraction

The titles and abstracts of the articles were screened according to the pre-set inclusion and exclusion criteria. Eligible studies were included after reading the full texts of the articles. Two researchers screened the literature individually without disturbing each other, and discrepancies were resolved by a third researcher. The following information was collected and extracted by two reviewers: author, publication year, type of fluids used, sample size, age, sex, and fluid dosage form and dosage. The outcome indicators included the rates of mortality, transfusion, atrial fibrillation, and AKI, urine volume within 24 h after surgery, postoperative blood loss, postoperative chest tube output over the first 24 h after surgery, the length of ICU stay, and the length of hospital stay.

### Quality evaluation

Quality evaluation was independently performed by two researchers. The Cochrane Collaboration’s tool, which included seven indicators categorized into a “low risk of bias”, an “unclear risk of bias” and a “high risk of bias”, was employed for assessing the risk of bias (Higgins et al. 2011).

### Assessment of quality of body of evidence

Grading of Recommendations Assessment, Development and Evaluation (GRADE) approach was applied to assess the quality of evidence. Inconsistency, risk of bias, indirectness, imprecision and publication bias was judged by two researchers working independently. Overall quality was deemed very low, low, moderate, or high using GRADE tool (GRADE Working Group) (Guyatt et al. 2011).

### Statistical analysis

We used risk ratios (RRs) with 95% confidence intervals (CIs) (Deeks 2002) to evaluate dichotomized data and mean differences (MDs) with 95% CIs (Higgins et al. 2001) to evaluate continuous data. For heterogeneity (Jackson 2006), the I^2^ value was used for all outcomes. When the I^2^ value was > 40%, a random effects model was adopted. Otherwise, a fixed-effects model was used. To further consider heterogeneity, subgroup analyses, including the fluid management time and type of crystalloid and colloidal solutions, were performed. In order to explore the difference of the dosage of colloids in the perioperative period, sensitivity analysis was used in this study to explore the stability of results. RevMan 5.4 software was used for data and statistical analyses.

## Results

### Literature identification

**Fig. 1:**
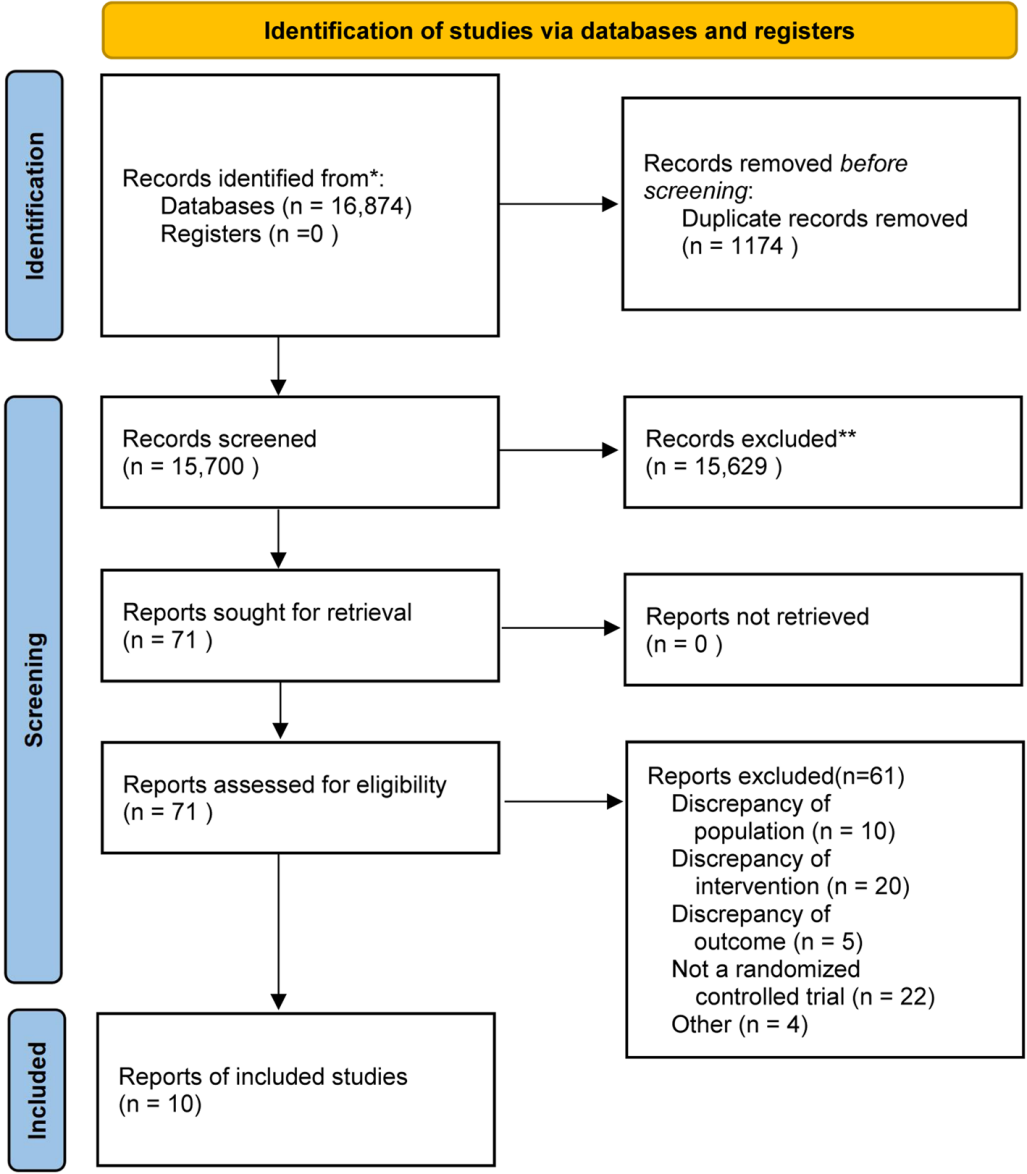
PRISMA flow diagram

Our research identified 16,874 records from all databases, and 1,174 duplicate studies were eliminated. 15,700 records were excluded by screening the titles. The full texts of 71 studies were screened. Ultimately, ten RCTs (Alavi et al. 2012; Boom et al. 2013; Lomivorotov et al. 2014; Magder et al. 2010; Schramko et al. 2010; Sirvinskas et al. 2007; Skhirtladze et al. 2014; Mazhar et al. 1998; Pesonen et al. 2022; Nagaya et al. 2022) were involved in this meta-analysis (Fig. 1). No other searches were conducted using other sources.

### Basic characteristics of the included studies

The meta-analysis included 2288 patients in ten RCTs. The characteristics of the included studies were summarized in Table 1, including the publication year, sample size, fluid dosage form and dosage, number of males and females, and age range of the sample. The subjects of the ten RCTs included middle-aged and elderly individuals. The crystalloid group was the control group, and the colloid group was the intervention group.

**Table 1:** Basic characteristics and demographics of the included studies

Study	Year	Sample	Age (year)	Gender (M/F)	Fluids	Fluid dosage form and dosage
Mazhar	1997	10	57.1 ± 7.8	10/0	7.2% Saline solution	7.2% Saline solution
		10	60.1 ± 7.8	7/3	Gelatin	Gelatin
Sirvinskas	2007	40	62.1 ± 9.8	/	6% HES	250 ml of a hypertonic solution of NaCl 7.2% in hydroxyethyl starch 6% solution
		40	65.6 ± 8.0	/	Ringer's solution	500 ml of the Ringer’s acetate solution
Magder	2010	118	65.5 ± 10.6	19/3	Hydroxyethyl Starch	250-mL Molecular weight 10% solution of pentastarch
		117	65.9 ± 10.6	18/4	0.9% NaCl	250-mL Boluses of 0.9% saline
Schramko	2010	15	65.0 ± 11	10/5	6% HES 130/0.4	6% HES 130/0.4 7 ml/kg over the following 12 h
		15	63.3 ± 8.0	9/6	4% Gelatin	4% Gelatin 7 ml/kg over the following 12 h
		15	64.5 ± 7.8	10/5	Ringer’s acetate solution	Ringer’s acetate solution 7 ml/kg over the following 12 h
Alavi	2012	29	59 ± 11	/	Ringer's solution	A maximum study drug dose of 50 mL/kg within 24 h
		31	60 ± 8.7	/	4% Gelatin	A maximum study drug dose of 50 mL/kg within 24 h
		32	57 ± 10.4	/	6% HES	A maximum study drug dose of 50 mL/kg within 24 h
Boom	2013	48	56.00 ± 6.57	/	6% HES	6% Hydroxyethyl starch
		50	56.49 ± 8.42	/	Hyperosmolar sodium lactate	Hyperosmolar sodium lactate
Lomivorotov	2014	20	58.6 ± 7.2	15/5	6% HES 200/0.5	6% Hydroxyethyl starch 4 mL/kg for 30 min
		20	56.2 ± 9.6	19/1	0.9% NaCl	0.9% NaCl 4 mL/kg for 30 min
Skhirtladze	2014	81	65.8 ± 12.2	52/29	6% HES 130/0.4	6%HES 130/0.4 up to 50 ml.kg/day
		76	64.4 ± 12.9	53/23	5% Albumin	5% Albumin up to 50 ml.kg/day
		79	65.5 ± 13.0	61/18	Ringer’s lactate	Ringer’s lactate up to 50 ml.kg/day
Nagaya	2022	28	65 ± 11	20/8	6% HES 130/0.4	The mean dose of 6% HES 130/0.4 was 28 ml/kg
		28	75 ± 11	14/14	Crystalloid solution	Crystalloid solution
Pesonen	2022	693	65 ± 10	549/144	4% albumin solution	2145 ± 178 ml 4% albumin solution
		693	65 ± 10	542/151	Ringer acetate solution	3292 ± 133 ml Ringer acetate solution

*HES* Hydroxyethyl starch, *M* Male, *F* Female, *NA* Not available

### Quality evaluation of the included studies

**Fig. 2:**
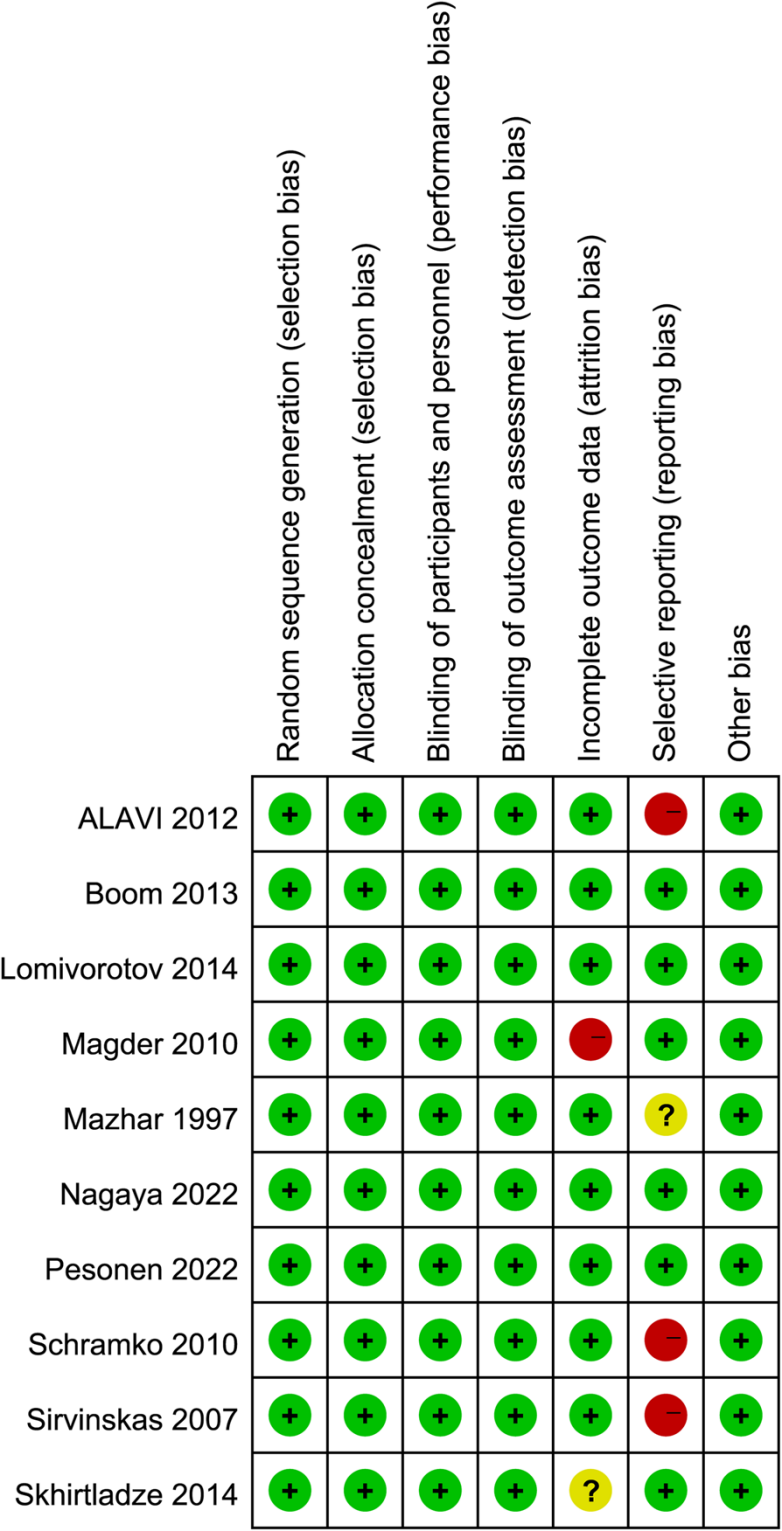
Quality evaluations of randomized controlled trials

The Cochrane Collaboration tool was employed for the quality evaluation of the ten RCTs, as shown in Fig. 2. The included studies were in line with the quality requirements of the analysis. There was an incomplete outcome data bias in one study (Magder et al. 2010). Three studies (Alavi et al. 2012; Schramko et al. 2010; Sirvinskas et al. 2007) had a certain selective reporting bias, and none of the included studies had other biases.

### Outcome indicators

#### Mortality

**Fig. 3:**
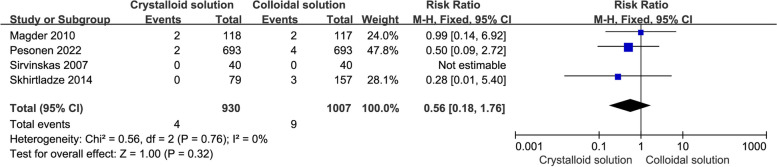
Comparison of mortality between colloid and crystalloid groups

Four RCTs (Magder et al. 2010; Sirvinskas et al. 2007; Skhirtladze et al. 2014; Pesonen et al. 2022) reported mortality, and the rate of death in one study (Sirvinskas et al. 2007) was zero (n_mortality_ = 0). Compared to crystalloids, colloids were not found to reduce mortality (*n* = 4, RR: 0.56, 95% CI: 0.18 to 1.76, *P* = 0.32), as shown in Fig. 3.

Furthermore, there was no statistically significant difference in mortality in the subgroup analysis, including iso-osmia crystalloid use (*n* = 4, RR: 0.56, 95% CI: 0.18 to 1.76, *P* = 0.32), HES use (*n* = 3, RR: 0.72, 95% CI: 0.14 to 3.60, *P* = 0.68), albumin use (*n* = 2, RR: 0.38, 95% CI: 0.09 to 1.62, *P* = 0.19), and postoperative use in the ICU (*n* = 2, RR: 0.99, 95% CI: 0.14 to 6.92, *P* = 0.99), as shown in Table 2.

**Table 2:** Results of subgroup analysis for all outcomes

Outcomes		Subgroup analysis		N	Sample	RR/MD, 95%CI	P for I^2^	I^2^	P for RR/MD
**Mortality**		**Total**		4	930/1007	0.56(0.18 to 1.76)	0.76	0%	0.32
		**Type of crystalloid**	**Iso-osmia**	4	930/1007	0.56(0.18 to 1.76)	0.76	0%	0.32
		**Fluid management time**	**ICU**	2	158/157	0.99(0.14 to 6.92)	NA	NA	0.99
		**Type of colloid**	**HES**	3	237/238	0.72(0.14 to 3.60)	0.57	0%	0.68
			**Albumin**	2	772/769	0.38(0.09 to 1.62)	0.59	0%	0.19
**Acute kidney injury**		**Total**		4	858/859	0.86(0.55 to 1.36)	0.76	0%	0.52
		**Type of crystalloid**	**Iso-osmia**	2	810/811	0.79(0.44 to 1.43)	0.86	0%	0.44
		**Type of colloid**	**HES**	3	165/166	1.00(0.51 to 1.97)	0.64	0%	1.00
			**Albumin**	1	693/693	0.78(0.43 to 1.44)	NA	NA	0.43
**Urine volume 24 h after surgery(mL)**		**Total**		4	184/275	-203.82(-689.61 to 281.96)	0.0001	86%	0.41
		**Type of crystalloid**	**Iso-osmia**	3	134/227	-373.86(-1024.33 to 276.61)	0.0006	86%	0.26
			**Hyper-tonic**	1	50/48	236.00(-33.24 to 505.24)	NA	NA	0.09
		**Fluid management time**	**ICU**	2	55/70	-667.43(-1165.00 to -169.86)	0.14	53%	0.009
			**Pre-CPB**	1	50/48	236.00(-33.24 to 505.24)	NA	NA	0.09
		**Type of colloid**	**HES**	4	184/184	-197.73(-710.00 to 314.53)	0.0001	85%	0.45
			**Gelatin**	1	15/15	-414.00(-1063.77 to 235.77)	NA	NA	0.21
**Postoperative blood loss(mL)**		**Total**		4	224/257	-104.08(-211.55 to 3.40)	0.02	69%	0.06
		**Type of crystalloid**	**Iso-osmia**	2	146/181	-66.92(-152.81 to 18.97)	0.19	42%	0.23
			**Hyper-tonic**	1	50/48	75.00(-261.77 to 411.77)	NA	NA	0.66
		**Fluid management time**	**ICU**	2	146/181	-66.92(-152.81 to 18.97)	0.19	42%	0.23
			**Pre-CPB**	1	50/48	75.00(-261.77 to 411.77)	NA	NA	0.66
		**Type of colloid**	**HES**	4	224/241	-91.89(-215.03 to 31.25)	0.006	76%	0.14
			**Gelatin**	1	29/31	-50.00(-184.12 to 84.12)	NA	NA	0.46
**postoperative chest tube output(mL)**		**Total**		4	827/920	-89.05(-165.75 to -12.36)	0.08	56%	0.02
		**Type of crystalloid**	**Iso-osmia**	4	827/920	-89.05(-165.75 to -12.36)	0.08	56%	0.02
		**Fluid management time**	**ICU**	2	55/70	1.45(-106.92 to 109.81)	0.29	11%	0.98
		**Type of colloid**	**HES**	3	134/136	-15.81(-99.40 to 69.04)	0.55	0%	0.72
			**Gelatin**	1	15/15	-178.00(-454.34 to 98.35)	NA	NA	0.21
**Transfusion type**	**Red blood cell**	**Total**		3	152/168	0.78(0.53 to 1.14)	0.89	0%	0.20
		**Type of crystalloid**	**Iso-osmia**	2	132/148	0.77(0.53 to 1.14)	0.67	0%	0.19
			**Hyper-tonic**	1	20/20	1.00(0.07 to 14.90)	NA	NA	1.00
		**Type of colloid**	**HES**	3	152/153	0.79(0.54 to 1.17)	0.71	0%	0.24
			**Gelatin**	1	15/15	0.75(0.20 to 2.79)	NA	NA	0.67
	**Fresh frozen plasma**	**Total**		3	152/168	0.57(0.30 to 1.08)	0.26	20%	0.08
		**Type of crystalloid**	**Iso-osmia**	2	132/148	0.50(0.26 to 0.99)	NA	NA	0.05
			**Hyper-tonic**	1	20/20	2.00(0.20 to 20.33)	NA	NA	0.56
		**Fluid management time**	**ICU**	2	132/148	0.50(0.26 to 0.99)	NA	NA	0.05
		**Type of colloid**	**HES**	3	152/153	0.57(0.30 to 1.08)	0.26	20%	0.08
	**Platelets**	**Total**		2	132/148	0.90(0.42 to 1.93)	0.55	0%	0.79
		**Fluid management time**	**ICU**	2	132/148	0.90(0.42 to 1.93)	0.55	0%	0.79
		**Type of crystalloid**	**Iso-osmia**	2	132/148	0.90(0.42 to 1.93)	0.55	0%	0.79
**Length of intensive care unit stay (h)**		**Total**		4	245/358	1.24(-7.05 to 9.54)	0.03	67%	0.77
		**Type of crystalloid**	**Iso-osmia**	3	225/338	-1.36(-9.20 to 6.49)	0.06	64%	0.73
			**Hyper-tonic**	1	20/20	15.40(-1.05 to 31.85)	NA	NA	0.07
		**Fluid management time**	**ICU**	2	146/188	-0.21(-2.15 to 1.73)	0.68	0%	0.83
			**Pre-CPB**	1	20/20	15.40(-1.05 to 31.85)	NA	NA	0.07
		**Type of colloid**	**HES**	4	245/250	1.44(-8.01 to 10.88)	0.01	73%	0.77
**Length of hospital stay (days)**		**Total**		3	216/295	0.73(-1.10 to 2.55)	0.35	4%	0.44
		**Type of crystalloid**	**Iso-osmia**	2	196/275	2.60(-0.80 to 6.00)	0.52	0%	0.13
			**Hyper-tonic**	1	20/20	0.00(-1.98 to 1.98)	NA	NA	1.00
		**Type of colloid**	**HES**	3	216/219	0.83(-1.16 to 2.83)	0.33	11%	0.41

*N* Number of included studies, *RR* Risk ratio, *MD* Mean difference, *CI* Confidence interval, *HES* Hydroxyethyl starch, *ICU* Intensive care unit, *CPB* Cardiopulmonary bypass, *NA* Not available

#### Postoperative complications

**Fig. 4:**
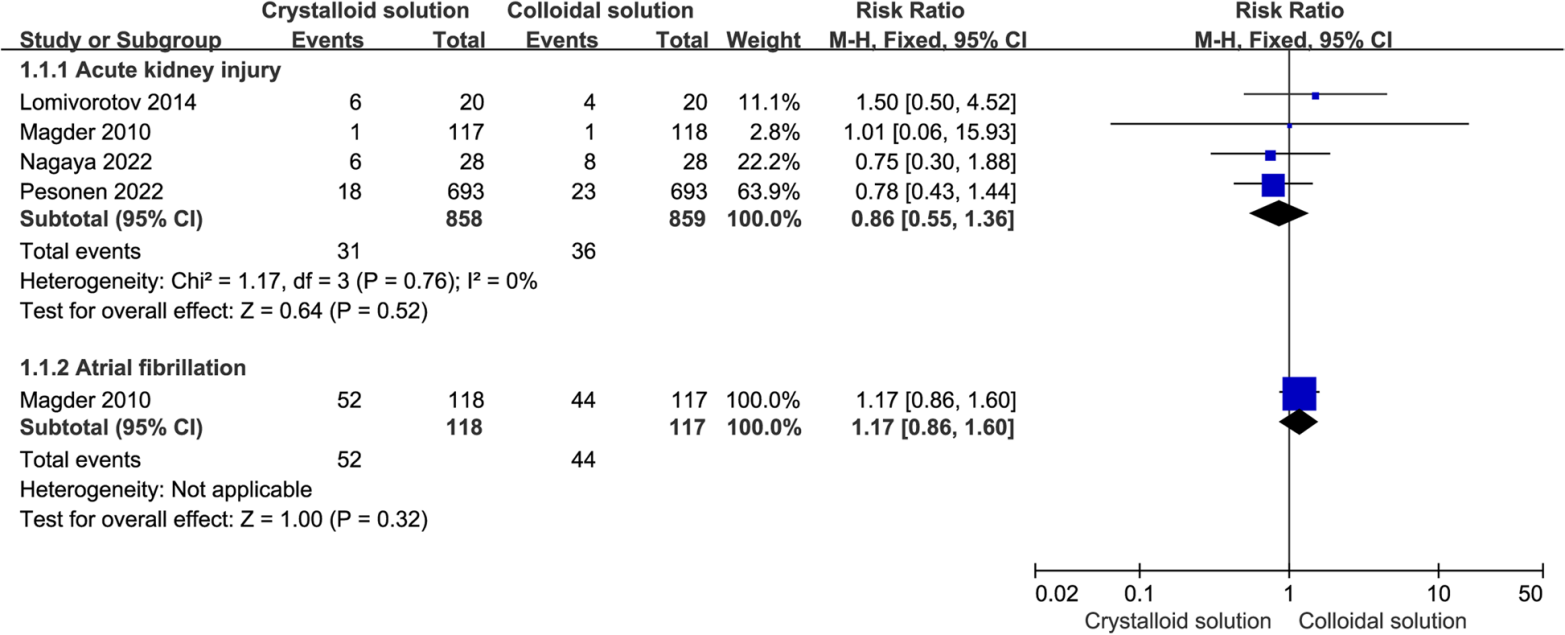
Comparison of postoperative complications (including those for acute kidney injury and atrial fibrillation) between colloid and crystalloid groups

A total of four RCTs (Lomivorotov et al. 2014; Magder et al. 2010; Pesonen et al. 2022; Nagaya et al. 2022) with 1717 patients reported postoperative AKI. Compared to crystalloids, colloids were not found to reduce the incidence of AKI (*n* = 4, RR: 0.86, 95% CI: 0.55 to 1.36, *P* = 0.52), as shown in Fig. 4.

Furthermore, there was no statistically significant difference in mortality in the subgroup analysis, including iso-osmia crystalloid use (*n* = 2, RR: 0.79, 95% CI: 0.44 to 1.43, *P* = 0.44), HES use (*n* = 3, RR: 1.00, 95% CI: 0.51 to 1.97, *P* = 1.00), and albumin use (*n* = 1, RR: 0.78, 95% CI: 0.43 to 1.44, *P* = 0.43), as shown in Table 2.

One study (Magder et al. 2010), including 235 patients undergoing cardiac surgery, reported atrial fibrillation outcome indicators. Compared to crystalloids, colloids were not found to reduce the risk of atrial fibrillation (*n* = 1, RR: 1.17, 95% CI: 0.86 to 1.60, *P* = 0.32), as shown in Fig. 4. Few studies reported atrial fibrillation, so no subgroup analysis was performed.

#### Urine volume 24 h after surgery

**Fig. 5:**
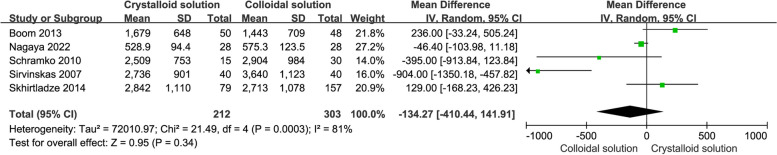
Comparison of urine volume within 24 h after surgery between colloid and crystalloid groups

Four RCTs (Boom et al. 2013; Schramko et al. 2010; Sirvinskas et al. 2007; Skhirtladze et al. 2014) involving 459 patients reported postoperative urine volume 24h after surgery. Compared to crystalloids, colloids were not found to increase the postoperative urine volume (*n*=4, MD: -203.82, 95% CI: -689.61 to 281.96, *P*=0.41), as shown in Fig. 5.

Furthermore, there was no statistically significant difference in urine volume in the subgroup analysis in Table 2, including iso-osmia crystalloid use (*n*=3, MD: -373.86, 95% CI: -1024.33 to 276.61, *P*=0.26), hypertonic crystalloid use (*n*=1, MD: 236.00, 95% CI: -33.24 to 505.24, *P*=0.09), pre-CPB use (*n*=1, MD: 236.00, 95% CI: -33.24 to 505.24, *P*=0.09), HES use (*n*=4, MD: -197.73, 95% CI: -710.00 to 314.53, *P*=0.45), and gelatine use (*n*=1, MD: -414.00, 95% CI: -1063.77 to 235.77, *P*=0.21). However, for postoperative fluid management in the ICU (*n*=2, MD: -667.43, 95% CI: -1165.00 to -169.86, *P*=0.009), patients in the colloid group had a significant increase in urine volume 24h after surgery.

#### Postoperative blood loss

**Fig. 6:**
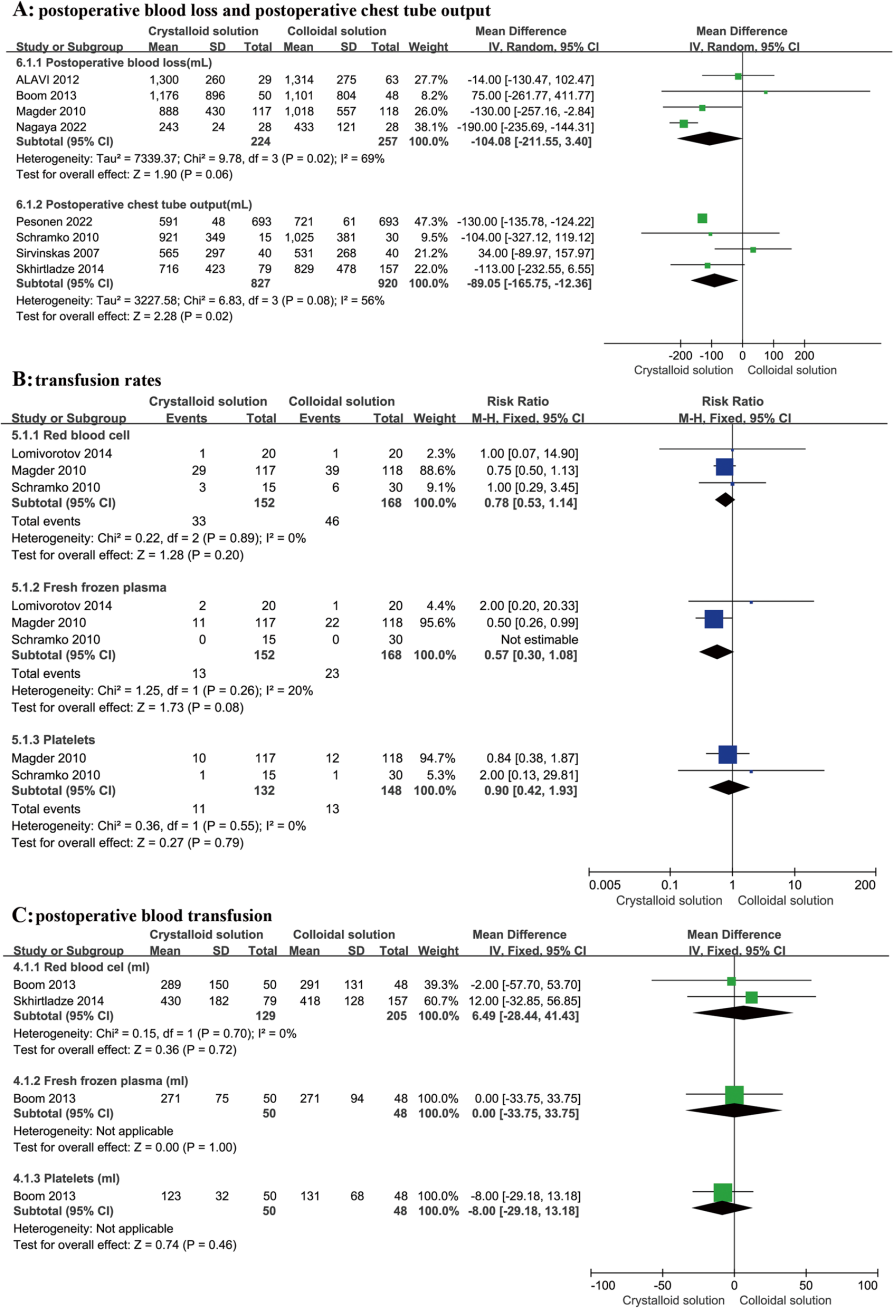
Comparison of postoperative blood loss, postoperative chest tube output, transfusion rates, and postoperative blood transfusion between colloid and crystalloid groups. Note: **A** indicates postoperative blood loss and postoperative chest tube output, **B** indicates transfusion rates (including those for red blood cell, fresh frozen plasma, and platelets), and **C** indicates postoperative blood transfusion

A total of four RCTs (Alavi et al. 2012; Boom et al. 2013; Magder et al. 2010; Nagaya et al. 2022) involving 481 patients reported an association between infusion type and postoperative blood loss. The results showed no correlation between postoperative blood loss and infusion type (*n* = 4, MD: -104.08, 95% CI: -211.55 to 3.40, *P* = 0.06), as shown in Fig. 6A. However, there appeared to be a lower trend in postoperative blood loss in the crystalloid group.

Furthermore, there was no statistically significant difference in postoperative blood loss in the subgroup analysis, as shown in Table 2, including iso-osmia crystalloid use (*n* = 2, MD: -66.92, 95% CI: -152.81 to 18.97, *P* = 0.23), hypertonic crystalloid use (*n* = 1, MD: 75.00, 95% CI: -261.77 to 411.77, *P* = 0.66), postoperative use in the ICU (*n* = 2, MD: -66.92, 95% CI: -152.81 to 18.97, *P* = 0.23), pre-CPB use (*n* = 1, MD: 75.00, 95% CI: -261.77 to 411.77, *P* = 0.66), HES use (*n* = 4, MD: -91.89, 95% CI: -215.03 to 31.25, *P* = 0.14), and gelatine use (*n* = 1, MD: -50.00, 95% CI: -184.12 to 84.12, *P* = 0.46).

It was observed that the crystalloid group had a certain tendency of reduced postoperative blood loss, so this needed to be further studied in high-quality and large sample RCTs.

#### Postoperative chest tube output

A total of four RCTs (Schramko et al. 2010; Sirvinskas et al. 2007; Skhirtladze et al. 2014; Pesonen et al. 2022) involving 1747 patients recorded the postoperative chest tube output. The results showed a statistically significant difference between the crystalloid group and the colloid group (*n* = 4, MD: -89.05, 95% CI: -165.75 to -12.36, *P* = 0.02), as shown in Fig. 6A, and the crystalloid group had significantly reduced postoperative chest tube output compared to the colloid group.

In the subgroup analysis, there was no statistically significant difference in postoperative chest tube output in the subgroup analysis, as shown in Table 2, including postoperative chest tube output in the ICU (*n* = 2, MD: 1.45, 95% CI: -106.92 to 109.81, *P* = 0.98), HES (*n* = 3, MD: -15.81, 95% CI: -99.40 to 69.04, *P* = 0.72), and gelatine (*n* = 1, MD: -178.00, 95% CI: -454.34 to 98.35, *P* = 0.21). However, the results showed that there was statistical significance regarding iso-osmia crystalloids (*n* = 4, MD: -89.05, 95% CI: -165.75 to -12.36, *P* = 0.02), and the crystalloid group had reduced postoperative chest tube output compared to the colloid group.

#### Transfusion rate

There were a total of three RCTs (Lomivorotov et al. 2014; Magder et al. 2010; Schramko et al. 2010) reporting on blood transfusion rates. To obtain more reasonable results, the analysis was divided into three subgroups according to the different blood products given to patients: the RBC, FFP, and platelet transfusion groups. Although postoperative transfusion was independent of infusion type, the RBC transfusion rate and FFP transfusion rate in the crystalloid group seemed to be lower, as shown in Fig. 6B, which needed to be further studied in high-quality and large sample RCTs.

A total of three RCTs (Lomivorotov et al. 2014; Magder et al. 2010; Schramko et al. 2010) involving 320 patients reported the postoperative RBC transfusion rate. Compared to crystalloids, colloids were not found to reduce the RBC transfusion rate (*n* = 3, RR: 0.78, 95% CI: 0.53 to 1.14, *P* = 0.20), as shown in Fig. 6B. Furthermore, there was no statistically significant difference in the subgroup analysis, as shown in Table 2, including iso-osmia crystalloid use (*n* = 2 RR: 0.77, 95% CI: 0.53 to 1.14, *P* = 0.19), hypertonic crystalloid use (*n* = 1, RR: 1.00, 95% CI: 0.07 to 14.90, *P* = 1.00), HES use (*n* = 3, RR: 0.79, 95% CI: 0.54 to 1.17, *P* = 0.24), and gelatine use (*n* = 1, RR: 0.75, 95% CI: 0.20 to 2.79, *P* = 0.67).

In addition, two RCTs (Boom et al. 2013; Skhirtladze et al. 2014) reported the average RBC transfusion volume for a total of 334 patients undergoing cardiac surgery, but there was no statistically significant difference in the RBC transfusion rate (*n* = 2, MD: 6.49, 95% CI: -28.44 to 41.43, *P* = 0.72) after surgery with the use of colloids and crystalloids, as shown in Fig. 6C.

Three articles (Lomivorotov et al. 2014; Magder et al. 2010; Schramko et al. 2010) reported the postoperative FFP transfusion rate, but the postoperative FFP transfusion rate from one study (Schramko et al. 2010) was zero. Compared to crystalloids, colloids were not found to reduce the risk of FFP transfusion (*n* = 3, RR: 0.57, 95% CI: 0.30 to 1.08, *P* = 0.08), as shown in Fig. 6B. Furthermore, there was no statistically significant difference in the subgroup analysis, including hypertonic crystalloid use (*n* = 1, RR: 2.00, 95% CI: 0.20 to 20.33, *P* = 0.56) and HES use (*n* = 3, RR: 0.57, 95% CI: 0.30 to 1.08, *P* = 0.08), as shown in Table 2. However, the results showed that there was a statistically significant difference in iso-osmia crystalloid use (*n* = 2, RR: 0.50, 95% CI: 0.26 to 0.99, *P* = 0.05) and postoperative use in the ICU (*n* = 2, RR: 0.50, 95% CI: 0.26 to 0.99, *P* = 0.05). The crystalloid group had a significantly reduced postoperative FFP infusion rate compared to the colloid group.

Two articles (Magder et al. 2010; Schramko et al. 2010) reported the postoperative platelet infusion rate. Compared to crystalloids, colloids were not found to reduce the risk of platelet infusion (*n* = 2, RR: 0.90, 95% CI: 0.42 to 1.93, *P* = 0.79), as shown in Fig. 6B. Furthermore, there was no statistically significant difference in the subgroup analysis, including postoperative use in the ICU (*n* = 2, RR: 0.90, 95% CI: 0.42 to 1.93, *P* = 0.79) and iso-osmia crystalloid use (*n* = 2, RR: 0.90, 95% CI: 0.42 to 1.93, *P* = 0.79), as shown in Table 2.

#### Length of ICU stay

**Fig. 7:**
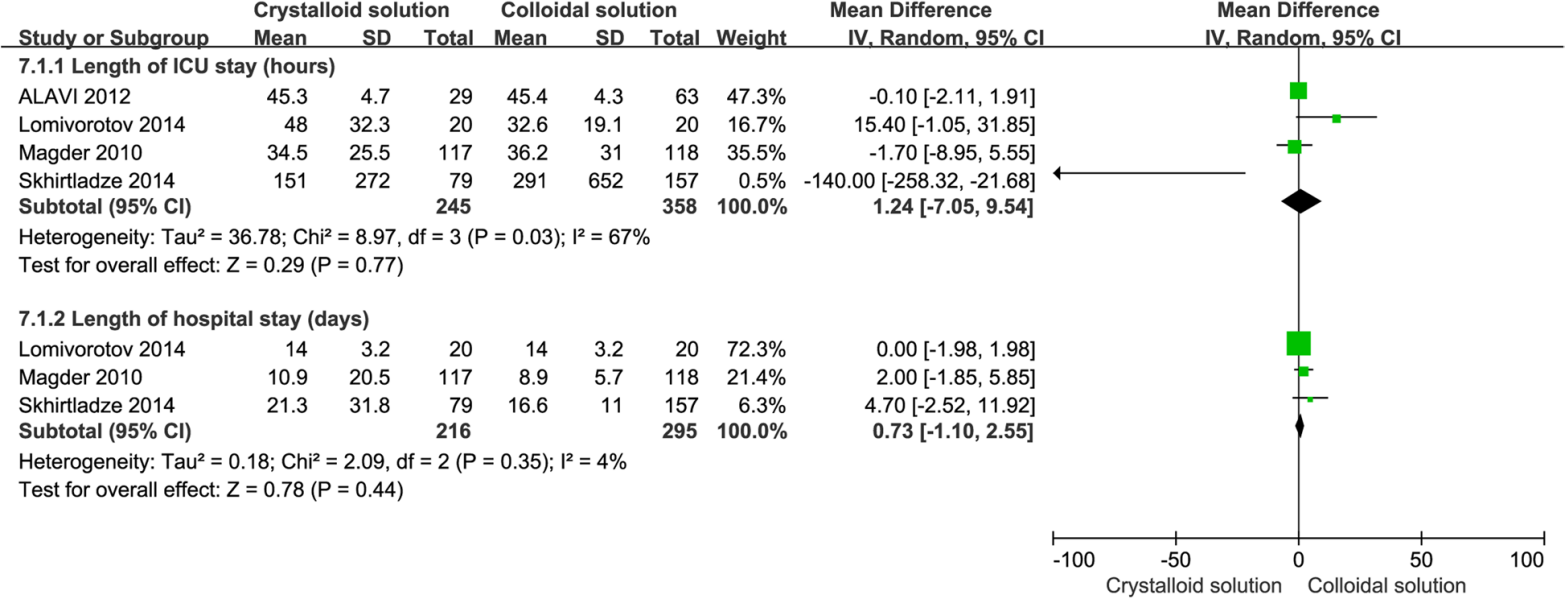
Comparison of length of ICU stay and length of hospital stay between colloid and crystalloid groups

Four RCTs (Alavi et al. 2012; Lomivorotov et al. 2014; Magder et al. 2010; Skhirtladze et al. 2014) reported the length of ICU stay. Compared to crystalloids, colloids were not found to reduce the risk of an increased length of ICU stay (*n* = 4, MD: 1.24, 95% CI: -7.05 to 9.54, *P* = 0.77), as shown in Fig. 7. Furthermore, there was no statistically significant difference in the subgroup analysis, including postoperative use in the ICU (*n* = 2, MD: -0.21, 95% CI: -2.15 to 1.73, *P* = 0.83), pre-CPB use (*n* = 1, MD: 15.40, 95% CI: -1.05 to 31.85, *P* = 0.07), iso-osmia crystalloid use (*n* = 3, MD: -1.36, 95% CI: -9.20 to 6.49, *P* = 0.73), hypertonic crystalloid use (*n* = 1, MD: 15.40, 95% CI: -1.05 to 31.85, *P* = 0.07), and HES use (*n* = 4, MD: 1.44, 95% CI: -8.01 to 10.88, *P* = 0.77), as shown in Table 2.

#### Length of hospital stay

Three RCTs (Lomivorotov et al. 2014; Magder et al. 2010; Skhirtladze et al. 2014) reported the length of hospital stay. Compared to crystalloids, colloids were not found to reduce the risk of an increased length of hospital stay (*n* = 3, MD: 0.73, 95% CI: -1.10 to 2.55, *P* = 0.44), as shown in Fig. 7. Furthermore, there was no statistically significant difference in the subgroup analysis, including iso-osmia crystalloid use (*n* = 2, MD: 2.60, 95% CI: -0.80 to 6.00, *P* = 0.13), hypertonic crystalloid use (*n* = 1, MD: 0.00, 95% CI: -1.98 to 1.98, *P* = 1.00), and HES use (*n* = 3, MD: 0.83, 95% CI: -1.16 to 2.83, *P* = 0.41), as shown in Table 2.

### Sensitivity analysis

**Fig. 8:**
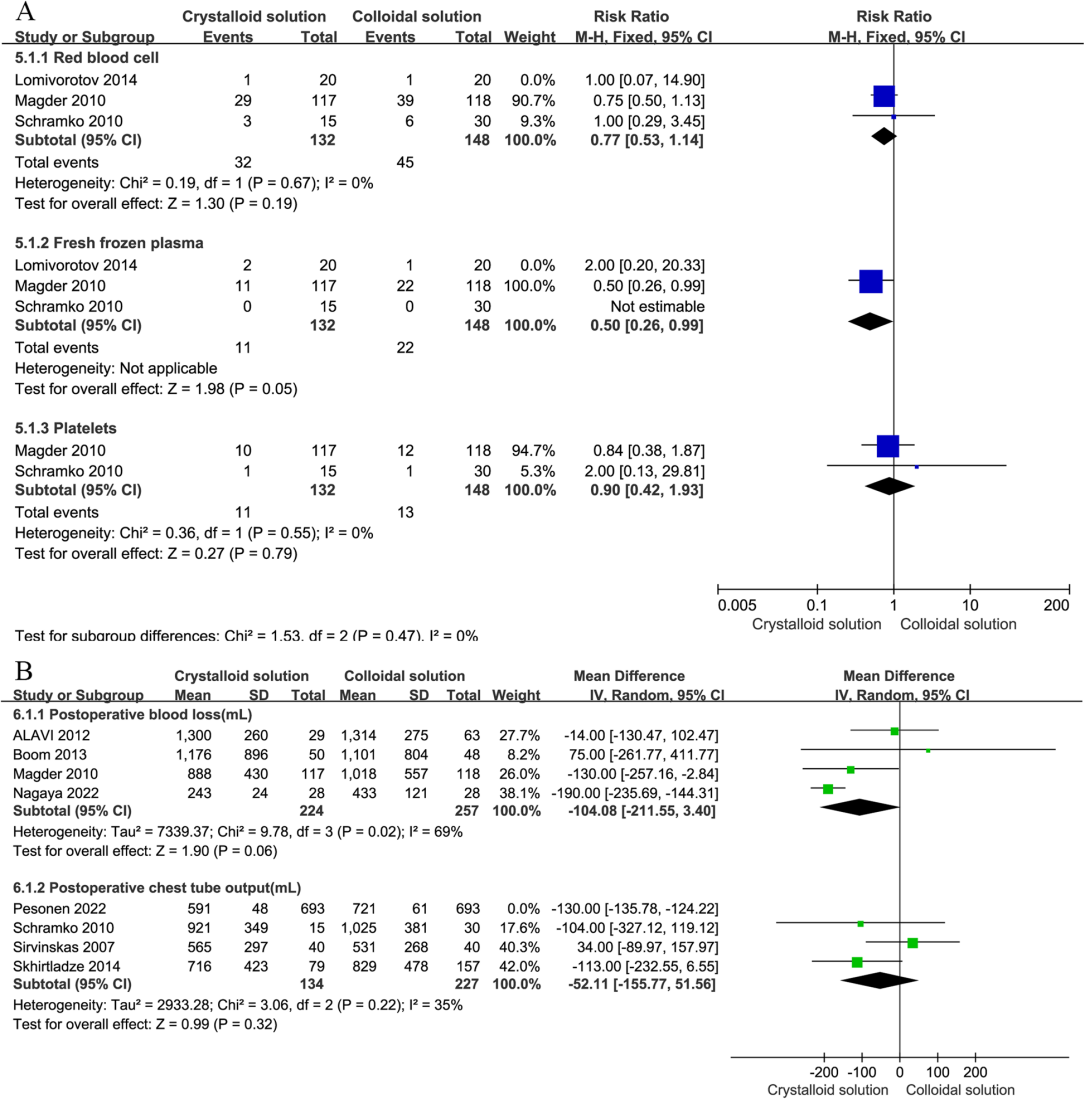
Forest of sensitivity analysis. Note: **A** indicates transfusion rates (including those for red blood cell, fresh frozen plasma, and platelets), and **B** indicates postoperative blood loss and postoperative chest tube output

In Lomivorotov et al.'s study (Lomivorotov et al. 2014), the usage of colloidal solution was 4 ml/kg, while in Pesonen et al.'s study (Pesonen et al. 2022), the maximum usage of colloidal solution could reach 3200 ml, indicating significant heterogeneity. The sensitivity analysis results showed that Lomivorotov et al.'s study had an impact on the FFP transfusion rate, and when this study was excluded, the results were statistically significant (*n* = 2, RR: 0.50, 95%CI: 0.26 to 0.99, *P* = 0.05) in Fig. 8A. When studying the postoperative chest tube output, Pesonen et al.'s study was excluded and there was no difference in the results (*n* = 3, MD: -52.11, 95% CI: -155.77 to 51.56, *P* = 0.32) in Fig. 8B.

### Grade evidence estimate

Mortality, AKI, and transfusion rate (RBC, FFP, and platelets) were assessed by the GRADE evidence estimate in Table 3. Mortality and AKI were downgraded by one level due to the difference in interventions included in the studies, which belonged to the moderate evidence. RBC was downgraded by two levels, the indirectness due to the differences in interventions between studies and the imprecision due to the small number of patients included. In terms of FFP, inconsistency, indirectness, and imprecision had been downgraded, and the evidence level was very low. Platelets were downgraded by two levels, the imprecision due to the small number of patients included and the inconsistency due to differences in point estimates between studies.

**Table 3:** GRADE profile of crystalloid solutions and colloidal solutions for cardiac surgery in mortality, AKI, and transfusion (RBC, FFP and platelet)

Quality assessment						Summary of findings			Quality of evidence
**Outcomes**	**Risk of bias**	**Inconsistency**	**Indirectness**	**Imprecision**	**Publication** **bias**	**Study event rates**			
						**With** **colloidal solution**	**With crystalloid solution**	**Relatives risk** **(95% CI)**	
**Mortality**	No serious limitations	No serious limitations	Serious limitations due to the indirectness^a^	No serious limitations	No serious limitations	9/1007 (0.9%)	4/930 (0.4%)	0.56 (0.18, 1.76)	⊕ ⊕ ⊕ ○ **Moderate**, Due to the indirectness
**AKI**	No serious limitations	No serious limitations	Serious limitations due to the indirectness^a^	No serious limitations	No serious limitations	36/859 (4.2%)	31/858 (3.6%)	0.86 (0.55, 1.36)	⊕ ⊕ ⊕ ○ **Moderate**, Due to the indirectness
**RBC**	No serious limitations	No serious limitations	Serious limitations due to the indirectness^a^	Serious limitations due to the imprecision^b^	No serious limitations	46/168 (27.4%)	33/152 (21.7%)	0.78 (0.53, 1.14)	⊕ ⊕ ○○ **Low**, Due to the indirectness and imprecision
**FFP**	No serious limitations	Serious limitations due to the inconsistency^c^	Serious limitations due to the indirectness^a^	Serious limitations due to the imprecision^b^	No serious limitations	23/168 (13.7%)	13/152 (8.6%)	0.57 (0.30, 1.08)	⊕ ○○○**Very Low**, Due to the inconsistency, indirectness and imprecision
**Platelet**	No serious limitations	Serious limitations due to the inconsistency^c^	No serious limitations	Serious limitations due to the imprecision^b^	No serious limitations	13/148 (8.8%)	11/132 (8.3%)	0.90 (0.42, 1.93)	⊕ ⊕ ○○ **Low**, Due to the inconsistency and imprecision

*AKI* acute kidney injury, *RBC* red blood cell, *FFP* fresh frozen plasma

^a^Differences in interventions from Lomivorotov et al.'s study and Pesonen et al.'s study lead to a decrease in credibility

^b^The small number of patients included leads to a decrease in credibility

^c^The differences in point estimates among different studies lead to a decrease in credibility

## Discussion

The main findings of this meta-analysis were that both colloids and crystalloids could be used as alternatives for perioperative fluid management in patients after cardiac surgery, and colloids were not superior to crystalloids as the choice of perioperative fluid infusion. The crystalloid group had significantly reduced postoperative chest tube output compared to the colloid group. For the use of isotonic crystalloids and crystalloids in the ICU after surgery, the rate of FFP infusion in the crystalloid group was lower than that in the colloid group. In addition, for fluid management in the ICU, patients in the colloid group had a significant increase in urine volume 24 h after surgery. No differences were found in mortality, length of ICU stay, length of hospital stay, postoperative blood loss, postoperative complications (AKI and atrial fibrillation), or the blood transfusion rate (RBCs and platelets) between the two groups. Thus, our analysis suggested that colloids and crystalloids might have similar effects in perioperative infusion in cardiac surgery patients.

Currently, approximately millions of people worldwide underwent cardiac surgery per year, and there was a lack of studies exploring all aspects of perioperative management, including patient and fluid management during the perioperative period, attracting the attention of clinical workers regarding the rational choice of fluid as a topic worthy of analysis. The inconclusive management of perioperative fluid involved postoperative blood transfusion, renal function, mortality, and other aspects of postoperative recovery (Lewis et al. 2018). The perioperative fluid infusion strategy, which was essential for patients during the perioperative period to achieve accelerated recovery, was explored comprehensively in this meta-analysis and systematic review and evaluated through various fluid management indices, such as the rates of mortality, transfusion, atrial fibrillation, and AKI, the length of ICU stay, urine volume within 24 h after surgery, postoperative blood loss, the length of hospital stay, and postoperative chest tube output over the first 24 h after surgery.

A study of non-cardiac surgery in adults showed that the application of colloids in goal directed fluid therapy was associated with a tendency to a higher mortality in patients who underwent surgery (Ripollés et al. 2015). There was a completely different idea that mortality in critically ill patients was not affected by fluid type, which was attributed to the fact that the haemodynamic stability of critically ill patients differs from that of patients undergoing cardiac surgery (Lewis et al. 2018). A meta-analysis (Skubas et al. 2024) evaluating cardiovascular surgery showed that colloidal solutions treatment did not reduce mortality in patients. Similar conclusions regarding the unclear effect of HES and albumin use on mortality in patients were demonstrated by a previous study (Bignami et al. 2017). However, further research was needed to explore which factor played the leading role in these relationships.

For postoperative transfusion rates, there were no direct or obvious differences among the RBC, FFP, and platelet transfusion rates. The crystalloid group seemed to have a certain tendency toward reduced postoperative RBC and FFP infusion rates, resulting from a combination of various factors rather than a single factor. A sufficient intravenous fluid volume could effectively reduce the need for postoperative RBC transfusion. The use of colloids had been shown to increase transfusion rates with evidence from a study by Lewis et al. (2018), which was not consistent with the results of our subgroup analyses. Although using large doses of HES did not increase the transfusion rate in cardiac surgery patients (Kasper et al. 2003), it could become a substitute for gelatine in cardiac surgery patients (Linden et al. 2005) and a credible alternative to albumin for paediatric cardiac surgery patients (Miao et al. 2014). Existing uncertainties regarding the mortality and transfusion rates of critically ill patients receiving colloids or crystalloids still confused researchers and clinical workers (Lewis et al. 2018). A systematic review and meta-analysis on the effects of HES on postoperative blood loss and clotting showed that HES increased blood loss (Rasmussen et al. 2016). This conclusion was marked based on patients who underwent noncardiovascular surgery, while the population involved in this meta-analysis included patients undergoing cardiac surgery.

Several studies had suggested that 6% HES (130/0.4) did not increase the risk of renal failure after surgery (Vives et al. 2016; Béchir et al. 2013; Jo et al. 2019; Mertens zur Borg et al. 2008), while there were also literature reports that 10% HES significantly increased the incidence of AKI (Werner et al. 2018). The choice of colloids or crystalloids after surgery had no significant influence on the occurrence of postoperative atrial fibrillation. Several studies revealed that the use of colloids did not significantly reduce the rate of atrial fibrillation compared to the use of crystalloids (Voldby et al. 2018; Adnan and Yandrapalli 2020).

Based on this meta-analysis, the length of ICU stay and the length of hospital stay were not significantly different according to the postoperative fluid type. Related studies were identified as evidence of insignificant differences in length of ICU stay or length of hospital stay with the use of either colloids or crystalloids during surgery, for instance, the application of HES for cardiac surgery patients (Feldheiser et al. 2013; Ooi et al. 2009; Choi et al. 1999), which was consistent with this meta-analysis. At the start of cardiopulmonary bypass surgery, the use of colloidal priming solutions was recommended to prevent the initial decrease in colloidal osmotic pressure (Jansen et al. 1996). The degradation of gelatine-based colloids was relatively slow, and the volume expansion effect was maintained for 2–3 h, while the volume expansion effect of the crystalloid solution was shorter. Gelatine-based colloids were considered effective in improving haemodynamics and peripheral perfusion and optimizing the intravascular volume state of patients.

For subgroups, in a previous comparative study conducted in critically ill patients undergoing fluid management, it was found that the use of colloids (including albumin, HES, and gelatine) had no effect on patient mortality compared to the use of crystalloids (Lewis et al. 2018), which was consistent with our research findings. After cardiac surgery, patients undergoing fluid management in the ICU had more urine output during hypertonic crystalloid therapy, in addition, there was no difference in the length of ICU stay or length of hospital stay between the two groups, which was inconsistent with our research results (Pfortmueller et al. 2020). This error might be due to inconsistent sample sizes and intervention measures.

Despite every effort to minimize errors and deviations, there were some limitations in this meta-analysis. Firstly, the different severities of heart diseases might lead to different prognosis outcomes, but relevant information could not be directly obtained and could only be predetermined by different cardiac surgery. Secondly, regarding the timing of perioperative fluid therapy, the use in different studies was inconsistent and relatively mixed, which was not conducive to subgroup analysis, which might have impacted the outcome. Thirdly, in the original research, the proportion of colloidal solution used could not be directly obtained, and there was significant heterogeneity in the amount of colloidal solution used. sensitivity analysis had been conducted. The small sample size and limited inclusion of studies had led to inconsistent results. Finally, the small number of included studies involving CPB prevented this study from performing a subgroup analysis to explore the effect of CPB on the overall outcome. Therefore, more studies were needed to further explore the association between the perioperative fluid management strategy and postoperative recovery of patients undergoing cardiac surgery.

## Conclusion

Both colloids and crystalloids could be used as alternatives for perioperative fluid management in patients after cardiac surgery. The use of crystalloids significantly reduced the postoperative chest tube output compared to the use of colloids. The use of isotonic crystalloids or fluid management during the ICU stay significantly decreased the rate of FFP infusion. ICU patients in the colloid group had higher urine output 24 h after surgery. In addition, although the infusion method was not related to most outcomes, the RBC transfusion rate, the FFP transfusion rate and postoperative blood loss in the crystalloid group seemed to be lower, which needed to be further studied in high-quality and large-sample RCTs.

### Supplementary Information

Supplementary Material 1.

## Abbreviations

AKI: Acute kidney injury
CI: Confidence interval
FFP: Fresh frozen plasma
HES: Hydroxyethyl starch
ICU: Intensive care unit
RBC: Red blood cell
RCTs: Randomized controlled trials
RR: Risk ratio
