# Supplementary material for: Use of colloids and crystalloids for perioperative clinical infusion management in cardiac surgery patients and postoperative outcomes: a meta-analysis
Source: Perioper Med (Lond). 2024 Jul 24;13:83. doi: 10.1186/s13741-024-00445-0 (PMC11267693; doi:10.1186/s13741-024-00445-0)
Supplement: Supplementary file 1 — Supplementary Material 1. [file 13741_2024_445_MOESM1_ESM.docx]

**Supplementary Method 1** Search strategy

**1. Ovid MEDLINE(R) ALL 1946 to August 25, 2023**

1. Thoracic Surgery.mp.
2. Thoracic Surgical Procedures.mp.
3. Cardiac Surgical Procedures.mp.
4. exp *heart surgery
5. Cardiac Surgery.mp.
6. Coronary Artery Bypass.mp.
7. (heart OR cardiac).ti,ab.
8. #1 OR #2 OR #3 OR #4 OR #5 OR #6 OR #7
9. Plasma Volume.mp.
10. Fluid Therapy.mp.
11. Colloids.mp.
12. Rehydration Solutions.mp.
13. Isotonic Solutions.mp.
14. Serum.mp.
15. Plasma.mp.
16. Plasma Substitutes.mp.
17. Albumin.mp.
18. Serum Albumin.mp.
19. Hydroxyethyl Starch Derivatives.mp.
20. Hetastarch.mp.
21. HAES-steril.mp.
22. Hydroxyethyl Starch.mp.
23. Ringer Lactate.mp.
24. Hartmanns Solution.mp.
25. Priming.mp.
26. Prime.mp.
27. (colloid* OR hydrocolloid* OR crystalloid* OR albumin* OR albumen* OR plasma OR starch* OR dextran* OR gelofus* OR hemaccel* OR haemaccel* OR serum OR hetastarch OR isotonic OR ringer* OR gelatin* OR gentran* OR pentastarch* OR pentaspan* OR hartman OR sodium OR potassium OR saline OR prime OR priming OR Hetastarch OR Ringer).ti,ab.
28. ((Isotonic adj1 saline adj1 solution*) OR (Blood adj1 substitut*) OR (blood adj1 expan*) OR (plasma adj1 volume adj1 expan*) OR (volume adj1 expan*)).ti,ab.
29. #9 OR #10 OR #11 OR #12 OR #13 OR #14 OR #15 OR #16 OR #17 OR #18 OR #19 OR #20 OR #21 OR #22 OR #23 OR #24 OR #25 OR #26 OR #27 OR #28
30. #8 AND #29
31. Limit #30 to (english language and humans and randomized controlled trial)

**2. EMBase <1974 to August 25, 2023>**

1. ‘Thoracic Surgery’/exp
2. ‘Thoracic Surgical Procedures’/exp
3. ‘Cardiac Surgical Procedures’/exp
4. ‘Heart surgery’/exp
5. ‘Cardiac Surgery’/exp
6. ‘Coronary Artery Bypass’/exp
7. (heart OR cardiac):ti,ab
8. #1 OR #2 OR #3 OR #4 OR #5 OR #6 OR #7
9. ‘Plasma Volume’/exp
10. ‘Fluid Therapy’/exp
11. ‘Colloids’/exp
12. ‘Rehydration Solutions’/exp
13. ‘Isotonic Solutions’/exp
14. ‘Serum’/exp
15. ‘Plasma’/exp
16. ‘Plasma Substitutes’/exp
17. ‘Albumin’/exp
18. ‘Serum Albumin’/exp
19. ‘Hydroxyethyl Starch Derivatives’/exp
20. ‘Hetastarch’/exp
21. ‘HAES-steril’/exp
22. ‘Hydroxyethyl Starch’/exp
23. ‘Ringer Lactate’/exp
24. ‘Hartmanns Solution’/exp
25. ‘Priming’/exp
26. ‘Prime’/exp
27. (colloid* OR hydrocolloid* OR crystalloid* OR albumin* OR albumen* OR plasma OR starch* OR dextran* OR gelofus* OR hemaccel* OR haemaccel* OR serum OR hetastarch OR isotonic OR ringer* OR gelatin* OR gentran* OR pentastarch* OR pentaspan* OR hartman OR sodium OR potassium OR saline OR prime OR priming OR Hetastarch OR Ringer):ti,ab
28. #9 OR #10 OR #11 OR #12 OR #13 OR #14 OR #15 OR #16 OR #17 OR #18 OR #19 OR #20 OR #21 OR #22 OR #23 OR #24 OR #25 OR #26 OR #27
29. #8 AND #28
30. #29 AND [randomized controlled trial]/lim AND [english]/lim AND [humans]/lim AND ([embase]/lim OR [embase classic]/lim) AND [article]/lim

**3. Cochrane Central Register of Controlled Trials < August 25, 2023>**

1. MeSH descriptor: [Thoracic Surgery] explode all trees
2. MeSH descriptor: [Thoracic Surgical Procedures] explode all trees
3. MeSH descriptor: [Thoracic Surgery, Video-Assisted] explode all trees
4. MeSH descriptor: [Cardiac Surgical Procedures] explode all trees
5. MeSH descriptor: [Thoracic Surgery] explode all trees
6. MeSH descriptor: [Coronary Artery Bypass] explode all trees
7. (Heart or Cardiac):ti,ab
8. #1 or #2 or #3 or #4 or #5 or #6 or #7
9. MeSH descriptor: [Plasma Volume] explode all trees
10. MeSH descriptor: [Fluid Therapy] explode all trees
11. MeSH descriptor: [Colloids] explode all trees
12. MeSH descriptor: [Hetastarch] explode all trees
13. MeSH descriptor: [Hydroxyethyl Starch] explode all trees
14. MeSH descriptor: [Rehydration Solutions] explode all trees
15. MeSH descriptor: [Isotonic Solutions] explode all trees
16. MeSH descriptor: [Serum] explode all trees
17. MeSH descriptor: [Plasma] explode all trees
18. MeSH descriptor: [Plasma Substitutes] explode all trees
19. MeSH descriptor: [Albumins] explode all trees
20. MeSH descriptor: [Serum Albumin] explode all trees
21. MeSH descriptor: [Hartmanns Solution] explode all trees
22. (colloid* or hydrocolloid* or crystalloid* or albumin* or albumen* or plasma or starch* or dextran* or gelofus* OR hemaccel* or haemaccel* or serum or hetastarch or isotonic or ringer* or gelatin* or gentran* or pentastarch* or pentaspan* or hartman or sodium or potassium or saline or priming or prime):ti,ab
23. #9 or #10 or #11 or #12 or #13 or #14 or #15 or #16 or #17 or #18 or #19 or #20 or #22
24. #8 and #23 in Trials
